# A Central Role of Abscisic Acid in Stress-Regulated Carbohydrate Metabolism

**DOI:** 10.1371/journal.pone.0003935

**Published:** 2008-12-12

**Authors:** Stefan Kempa, Julia Krasensky, Silvia Dal Santo, Joachim Kopka, Claudia Jonak

**Affiliations:** 1 Gregor Mendel Institute of Molecular Plant Biology, Austrian Academy of Sciences, Vienna, Austria; 2 Max Plank Institute of Molecular Plant Physiology, Golm, Germany; Purdue University, United States of America

## Abstract

**Background:**

Abiotic stresses adversely affect plant growth and development. The hormone abscisic acid (ABA) plays a central role in the response and adaptation to environmental constraints. However, apart from the well established role of ABA in regulating gene expression programmes, little is known about its function in plant stress metabolism.

**Principal Findings:**

Using an integrative multiparallel approach of metabolome and transcriptome analyses, we studied the dynamic response of the model glyophyte *Arabidopsis thaliana* to ABA and high salt conditions. Our work shows that salt stress induces complex re-adjustment of carbohydrate metabolism and that ABA triggers the initial steps of carbon mobilisation.

**Significance:**

These findings open new perspectives on how high salinity and ABA impact on central carbohydrate metabolism and highlight the power of iterative combinatorial approaches of non-targeted and hypothesis-driven experiments in stress biology.

## Introduction

High soil salinity is a major environmental constraint for plant growth and development, and limits agronomic yield [1]. Salt stress affects almost all aspects of plant physiology. A decrease in water availability constitutes the primary limitation and leads to osmotic stress. In the second phase, high concentrations of sodium ions are toxic to the cell as they inhibit essential enzymes, cell division and expansion. Membranes may become disorganised and excess levels of reactive oxygen species (ROS) are produced, leading to oxidative stress [2]–[4].

Genetic and biochemical studies indicate that distinct signal transduction pathways mediate different aspects of high salt stress, which ultimately lead to reprogramming of metabolism, physiology and morphology [5]. Recovery of ion balance under salinity can be achieved by restriction of entry, vacuolar compartmentalisation and extrusion of sodium from the cell. For osmotic adjustment, plants can accumulate osmoprotective compounds such as proline, glycine betaine, sugars and sugar alcohols [6]. Additionally, compatible solutes appear to play a beneficial role by stabilising membranes and proteins, preserving the activity of enzymes and scavenging ROS [7].

Salinity has a strong impact on gene expression [8]–[11]. Salt stress-inducible genes comprise genes that directly protect cells from stress and genes that code for regulatory proteins. The first group includes genes involved in the synthesis of osmoprotectants, detoxification enzymes, ion channels and transporters, whereas the second group comprises genes encoding protein kinases and phosphatases, transcription factors, and enzymes required for the synthesis of the hormone abscisic acid (ABA).

ABA is an important regulator in many aspects of plant growth and development, and is pivotal for stress resistance [12]–[14]. Interestingly, ABA also plays a recently discovered role in hydroid regeneration, stress-adaptation in sponges, pathogenesis of *Toxoplasma gondii* and in human immune responses, thus indicating a conservation of ABA signalling across kingdoms [15]–[18]. In plants, ABA accumulates in response to different environmental stresses such as high salt, cold and drought. ABA-deficient plants have a modified tolerance to high salt conditions [19]–[21]. ABA appears to be perceived at several sites both extracellularly and intracellularly [22]–[24]. Phospholipids, heterotrimeric G proteins, modulation of intracellular calcium levels and the action of protein kinases and phosphatases are involved in ABA signalling [25], [26]. As an integral part of stress signal transduction, ABA regulates important cellular reactions such as stomatal closure and gene expression. Based on the observation that only a subset of stress-inducible genes responds to ABA, ABA-dependent and independent regulatory pathways have been suggested to mediate stress response [27].

Although global adjustment of metabolism is vital for adaptation to high salinity, our knowledge of the mechanisms involved in metabolic adaptation upon adverse environmental conditions is scarce. Early events largely determine whether plants can cope with stress [28] and point to the mechanisms plants use to adapt. Here, we investigated the metabolic response in comparison to the transcriptional reaction during the initial stages of salt stress in a time-dependent manner. Channelling of data from this comprehensive approach to targeted analyses allowed us to unravel the impact of the phytohormone ABA on metabolic regulation.

## Results and Discussion

### Metabolic dynamics in response to high salt conditions

Plant metabolism is highly flexible and has the potential to adjust to changing environmental conditions. To explore the temporal dynamics of the *Arabidopsis thaliana* metabolome in response to high soil salinity on a large scale, we performed metabolic profiling on leaves of plants exposed to high salt stress. Four week old soil-grown plants were watered with 150 mM NaCl and analysed 6 h, 12 h, 1 d, 3 d and 5 d after the onset of stress. While prolonged stress exposure led to sodium accumulation in leaves, the concentration of soluble sodium did not increase during the early experimental timepoints (Figure S1), indicating that the immediate metabolic modifications are not caused by ion toxicity.

**Figure 1:**
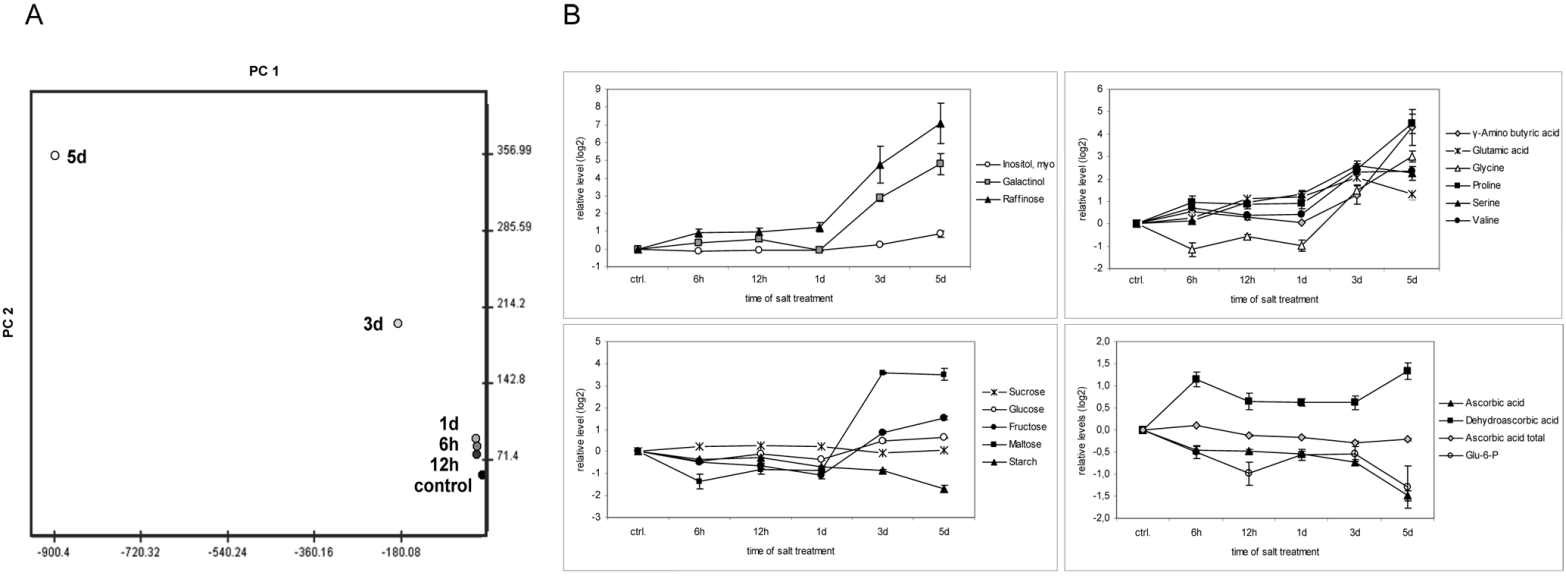
Temporal dynamics of the *Arabidopsis* metabolome upon salt stress. Metabolic profiling was performed on control plants and on plants exposed to high soil salinity for 6 h, 12 h, 24 h, 3 d and 5 d. (A) Principal component analysis (PCA) on the metabolic dynamics. PC1 gives a high weighting to raffinose, GABA, galactinol and proline; PC2 to glutamine, glutamic acid and G6P. (B) Relative metabolite levels of selected metabolites upon salt stress. Relative metabolite levels were log2 transformed and standard deviations were calculated from four independent measurements of pools consisting of ten plants each.

Comprehensive metabolic profiling was performed by gas chromatography-mass spectrometry (GC-MS) for polar compounds. Additionally, we determined the levels of starch, the main carbohydrate store in plants, and of ascorbate and dehydroascorbate, as parameters of cellular redox-balance. Principal component analysis (PCA) of the metabolic data revealed a continuous change of the metabolome upon high salt stress. We observed minor overall changes in metabolite levels early after application of high salt conditions but a pronounced alteration after prolonged stress (Figures 1A and S2 and Table 1 and S1) which is consistent with a recent analysis of *in vitro*-cultured *Arabidopsis* cells [29]. The clear separation of the 3 d and 5 d-metabolic profiles from the metabolome of unstressed plants highlights the plasticity of *Arabidopsis'* metabolism upon salt stress.

**Table 1:** High salinity-induced changes in metabolite levels.

*TAG RI*	*TAG MASS*	*REV MATCH*	*COMPOUND*	*ctrl.*	*6h*	*12h*	*1d*	*3d*	*5d*
1740.00	272	917	Aconitic acid	1.00	1.06	0.96	1.41	0.56	**0.37**
			Ascorbic acid	1.00	**0.73**	**0.72**	**0.69**	**0.60**	**0.36**
			Ascorbic acid total	1.00	1.08	0.91	0.89	0.82	0.86
1421.52	118	975	Aspartic acid	1.00	0.87	0.85	0.87	1.18	0.68
1804.48	147	980	Citric acid	1.00	**1.96**	**1.46**	**1.57**	1.11	0.84
			Dehydroascorbic acid	1.00	**2.21**	1.57	1.54	1.54	**2.52**
1259.77	156	958	Ethanolamine	1.00	**1.42**	1.02	0.90	0.99	1.42
1860.48	129	978	Fructose	1.00	0.72	**0.64**	**0.47**	**1.82**	**2.85**
1348.79	147	974	Fumaric acid	1.00	**1.80**	**1.59**	**1.26**	1.08	0.93
2961.58	117	965	Galactinol	1.00	1.28	1.49	0.97	**7.35**	**27.54**
1978.80	129	950	Galactonic acid	1.00	0.96	0.92	**0.80**	**0.75**	**0.79**
1983.47	129	948	Gluconic acid	1.00	**1.74**	**1.87**	1.25	**2.29**	**5.90**
1877.02	229	970	Glucose	1.00	**0.74**	0.93	**0.79**	**1.39**	**1.54**
2303.56	299	916	Glucose-6-phosphate	1.00	0.71	**0.50**	0.68	0.69	**0.41**
1528.27	158	944	Glutamic acid	1.00	1.20	**2.19**	**2.32**	**4.20**	**2.49**
1766.29	100	946	Glutamine	1.00	**2.14**	**0.32**	1.34	1.14	**2.96**
1338.96	147	956	Glyceric acid	1.00	1.00	0.99	0.96	0.78	0.82
1301.91	147	970	Glycine	1.00	**0.45**	**0.68**	**0.51**	**2.80**	**8.08**
1887.43	229	806	Indole-3-acetonitrile	1.00	**1.79**	**2.02**	**1.33**	**1.63**	**1.38**
2668.52	129	912	Lactose	1.00	1.08	0.88	0.83	1.09	0.76
1477.67	117	987	Malic acid	1.00	**1.98**	**1.74**	**1.52**	1.17	0.96
2715.89	160	973	Maltose	1.00	**0.39**	**0.57**	**0.55**	**12.04**	**11.41**
1914.96	147	960	Mannitol	1.00	**0.88**	1.05	1.05	1.00	0.91
2078.23	129	966	Myo-inositol	1.00	0.91	0.95	0.94	**1.21**	**1.79**
1555.80	146	955	Phenylalanine	1.00	**1.39**	**2.20**	**1.61**	**4.37**	**6.78**
1262.14	135	999	Phosphoric acid	1.00	**0.78**	**0.67**	**0.52**	**0.69**	**0.48**
1179.06	113	999	Proline	1.00	**1.94**	**1.85**	**1.88**	**5.41**	**22.10**
1521.02	156	981	Pyroglutamic acid	1.00	1.11	0.69	0.90	1.08	0.92
3344.18	204	936	Raffinose	1.00	**1.85**	**1.96**	**2.35**	**26.79**	**136.14**
1253.80	147	982	Serine	1.00	1.08	**1.92**	**2.47**	**5.97**	**4.76**
1792.80	204	894	Shikimic acid	1.00	0.92	0.81	**0.73**	0.78	**0.49**
2245.96	338	926	Sinapic acid. trans	1.00	**1.43**	1.09	0.95	1.29	1.04
2192.32	116	950	Spermidine	1.00	0.79	0.82	**0.39**	**0.32**	**0.23**
			Starch	1.00	**0.78**	**0.83**	**0.62**	**0.55**	**0.31**
1310.30	147	978	Succinic acid	1.00	1.35	1.22	1.16	**1.15**	1.42
2620.12	129	970	Sucrose	1.00	1.15	**1.21**	**1.17**	0.95	1.03
1545.59	147	967	Threonic acid	1.00	1.51	**1.54**	1.32	0.95	1.01
1373.34	101	968	Threonic acid-1.4-lactone	1.00	1.12	1.07	1.15	**0.74**	**0.71**
1290.44	130	982	Threonine	1.00	0.90	1.14	1.15	1.54	**1.37**
2722.90	143	994	Trehalose	1.00	0.73	**3.36**	1.09	**1.47**	**1.51**
1931.81	218	999	Tyrosine	1.00	**4.30**	**2.37**	**4.56**	**7.20**	**9.39**
2091.59	157	970	Uric acid	1.00	1.03	1.07	1.04	1.10	**1.28**
1209.27	144	968	Valine	1.00	**1.64**	1.32	1.32	**4.98**	**5.16**
1524.11	100	927	γ-Aminobutyric acid	1.00	1.46	1.23	1.05	**2.47**	**19.81**

The metabolite contents of rosette leaves of 4 week old, soil-grown *Arabidopsis thaliana* were determined upon watering with 150 mM NaCl. Pools of ten individual leaf rosettes were analysed four times using GC-MS. Compounds were identified by their retention index (TAG RI), quantitative mass tag (TAG MASS) and reverse match value (REV MATCH) of the mass spectra comparison. Ascorbic acid and dehydroascorbic acid levels were determined by HPLC and starch levels were quantified spectrophotometrically. Values are x-fold changes compared to the corresponding unstressed controls. Bold letters indicate significant changes of metabolites (t-test; P-value <0.05).

Analysis of individual metabolite levels showed that high salinity had a profound effect on central metabolism and induced complex changes in the metabolome in a time-dependent manner (Table 1 and Figure 1B). Temporal resolution revealed sustained and transient, early and late-responsive metabolic alterations. The early response to high salinity (6 to 12 h) was characterised by an increase in raffinose, trehalose, tyrosine, serine, phenylalanine, glutamic acid, proline, gluconic acid and dehydroascorbate, and a decrease in maltose, fructose, glucose-6-phosphate (G6P) and ascorbic acid levels. Prolonged stress (3 and 5 d) induced a strong accumulation of raffinose. Moreover, the levels of maltose, galactinol, proline and γ-amino butyric acid (GABA) were highly induced. Raffinose, galactinol and proline function as osmoprotectants and accumulate under stress conditions [7], [30]–[34]. GABA metabolism plays a major role in carbon and nitrogen metabolism. The strong increase in the amounts of GABA as a result of high salinity is consistent with its putative role in stress defence [35]–[37]. Upon long-lasting stress, the levels of amino acids including valine, tyrosine, serine, phenyalanine, glycine, glutamine and glutamic acid showed a coordinated increase which might be due to enhanced protein catabolism and/or re-allocation of nitrogen caused by growth inhibition. A similar accumulation of amino acids has been observed recently in *Lotus japonicus* and *Populus euphratica*, indicating a conservation of this response to high salinity [38], [39].

Redox balance is crucial for cellular function. Application of high salt stress did not change the overall pool size of the antioxidant ascorbate. However, a rapid and sustained increase in oxidised dehydroascorbate (Dha) and a decrease in reduced ascorbate (Asc), which led to a marked shift in the Asc to Dha ratio, was observed (Figure 1B). These data indicate that high salinity has an immediate early impact on the cellular redox state. Interestingly, the levels of G6P, which is a source of the redox equivalent NADPH under stress conditions [40], decreased in parallel with Asc.

High salt conditions induced a rapid alteration of carbohydrate levels. After irrigation of plants with NaCl, the levels of several soluble sugars and the glucose to fructose ratio changed significantly, indicating an impairment of central carbohydrate metabolism (Table S2). Notably, prolonged salt stress led to a substantial decrease in starch content and a strong increase in maltose (Table 1 and Figure 1B). Starch is the major carbohydrate store in plants and is important for energy and carbon skeleton supply [41]. In leaves, starch is degraded via the β-amylolytic pathway to maltose [42]–[44]. Maltose was also found to accumulate in *Lotus japonicus* [38] and starch levels were reported to decrease in tomato leaves [45] upon prolonged salt stress, suggesting that starch mobilisation might be at the heart of the change in carbohydrate metabolism in response to saline conditions. In general, a stress-induced increase in maltose levels, a reduction in the amount of G6P and a decrease in the ratio of glucose to fructose may reflect an altered carbon flux upon salt stress.

**Figure 2:**
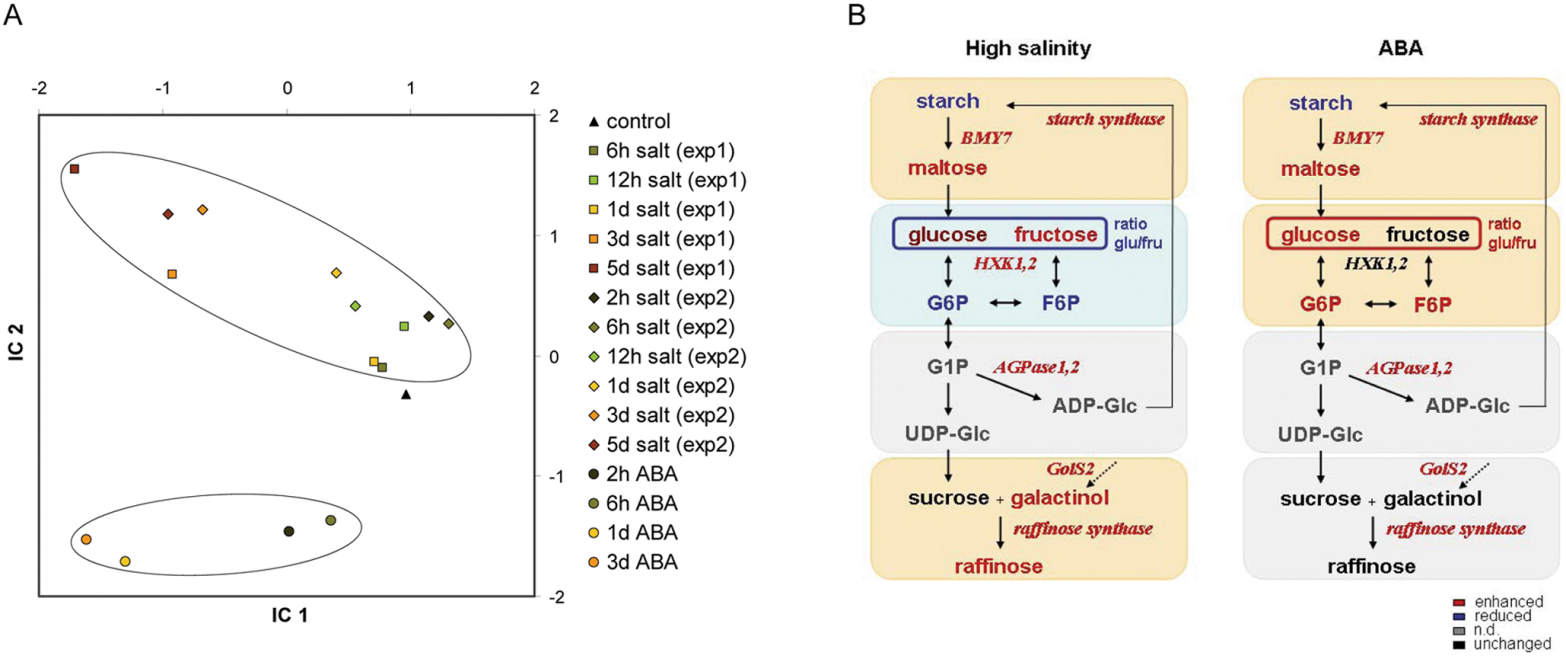
Common and distinct metabolic responses to high salinity and ABA. The metabolic response of 4 week old soil-grown *Arabidopsis* plants to high soil salinity and ABA was compared. (A) Independent component analysis (ICA) of metabolite data after salt stress and ABA treatment. IC1 gives high loadings to maltose, proline, phenylalanine, glutamine and glycine. IC2 gives high loadings to raffinose, galactinol and G6P. (B) Schematic representation of the starch to sucrose and RFO pathways after 3 days of treatment. Metabolites (bold) and genes (italic) whose levels were upregulated in comparison to controls are indicated in red whereas blue represents downregulation. Orange and blue shading indicate up- and downregulation of the modules within the metabolic pathways, respectively.

The dynamics of the overall changes in the metabolite composition induced by high soil salinity were confirmed in a second independent experiment (Figure 2A and Table S3).

### Transcriptional response of metabolism-related genes

To address the question of whether the observed changes in metabolite levels correlate with the transcriptional response of genes encoding the corresponding metabolic enzymes, we explored the *Arabidopsis* transcriptome. We employed the MapMan gene ontology and image annotator [46], [47] to examine the publicly available AtGeneExpress microarray data in order to identify metabolic genes that are significantly up- or downregulated by salt stress (Table S4). Numerous genes encoding isoforms of metabolic enzymes were found to be transcriptionally responsive to high salt stress. We focused our analyses on genes involved in starch metabolism, sugar conversion, the raffinose oligosaccharide (RFO) pathway and the ascorbate/glutathione cycle as the levels of metabolites involved in these pathways were significantly affected by salt treatment.

AtGeneExpress microarray analyses were performed on young hydroponically-grown plants whereas metabolic profiles were obtained from soil-grown adult plants. To verify the transcriptional response in our experimental system and for direct comparison of the transcriptional and metabolic salt-induced patterns, we performed semi-quantitative RT-PCR on 48 selected genes from the same plant material which was used for metabolic profiling. 75% of the monitored genes displayed a similar expression pattern upon high salinity treatment in young hydroponically-grown and in adult soil-grown plants (Table S4), emphasizing the robustness of the transcriptional response to high salt stress.

Starch metabolism is very sensitive to the environment [48]–[55]. We observed a depletion of starch and an important increase in maltose levels upon exposure of plants to high salinity. Consistently, beta-amylase (*BMY1* and *BMY7*), which cleaves maltose from starch-derived glucan molecules, was transcriptionally upregulated. Enhanced starch degradation by beta-amylase likely supplies energy and carbohydrate skeletons for adaptation processes (e.g. osmolyte production). Interestingly, genes involved in starch synthesis including an isoform of starch synthase and ADP-glucose pyrophosphorylase (*AGPase1* and *2*), which provides ADP-glucose, were also induced by high salinity. This observation might indicate that salt stress stimulates both starch mobilisation and starch synthesis. This interesting notion should be further addressed by metabolic flux analysis in future studies.

High salinity induced alterations in the levels of soluble sugars (Table 1 and Figure 1B). Several genes encoding enzymes in the sugar conversion pathway were found to be upregulated by salt stress. The expression of genes encoding glucose-6-phosphate dehydrogenase (*G6P DH*), invertase (*INV*), hexokinase (*HXK1* and *2*) and UDP-glucose pyrophosphorylase (*UGPase*) were induced to different extents. In line with the accumulation of galactinol and raffinose, genes coding for galactinol synthase (*GolS1, 2* and *3*) and raffinose synthase (*RS*) were strongly induced by high salt conditions.

Salt stress modified the expression of several genes encoding proteins involved in nitrogen metabolism. The majority of the monitored transcripts were strongly upregulated at later timepoints including pyrroline-5-carboxylate synthase (*P5CS1* and *2*), phenyl ammonium lyase (*PAL*), nitrate reductase (*Nry* and *NR*) and proline oxidase (*ERD5* and *PrOX*). Genes encoding different isoforms of glutamate decarboxylase (*GAD*) showed a diverse transcriptional response. GABA accumulated to high levels after 5 d in saline conditions. The expression of the glutamate decarboxylases GAD3 and GAD4 showed a biphasic induction whereas the expression of GAD2 decreased at later timepoints. The GABA transporters GABA trans1 and GABA trans2 (AtGAT1) were also induced transcriptionally. We observed a rapid change in the ascorbate to dehydroascorbate ratio by salt stress. However, with the exception of a glutathione reductase, genes of the ascorbate/glutathione cycle did not respond at the transcriptional level.

Taken together, metabolite levels and expression levels of genes encoding the corresponding metabolic enzymes showed co-regulation in some but not all of the pathways examined. The complex interrelation between gene expression and metabolite levels suggests that a multifaceted interplay at different control levels governs salt-induced metabolic re-adjustment.

### Correlation of the transcriptional and metabolic responses

**Figure 3:**
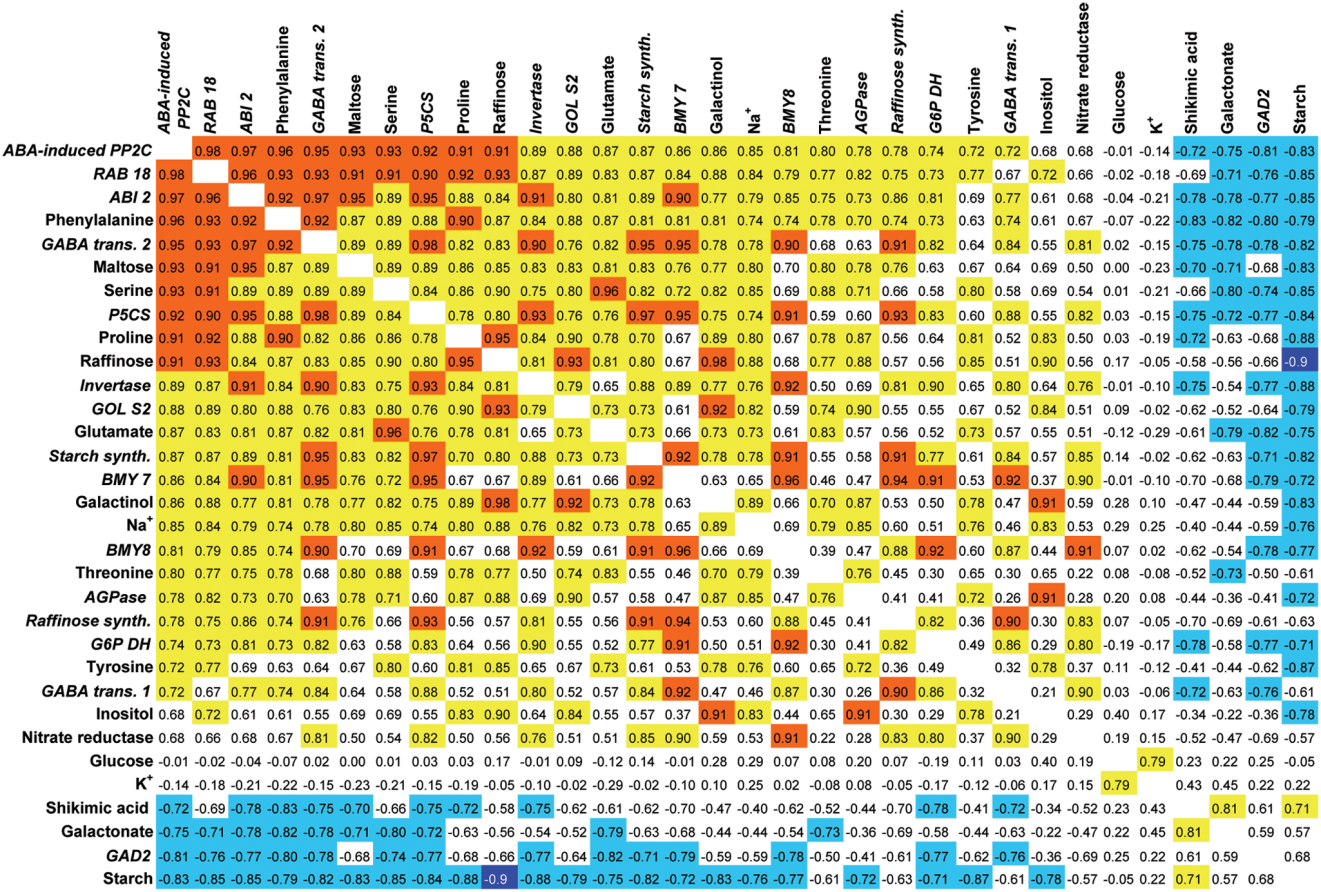
Correlation matrix of high salinity-induced metabolic and transcriptional changes. Pearson correlation coefficients of a pairwise correlation analysis of changes in metabolite levels and gene expression from all timepoints of two independent salt stress experiments. The background colours mark the value of the coefficient: orange: >0.91; yellow: >0.7; blue: <−0.7. Pearson correlation coefficients >0.91 and <−0.91 are significant (P-value <0.05) and correlation coefficients >0.7 and <−0.7 are suggested to be biologically relevant.

To compare changes in metabolite and transcript levels comprehensively, we generated a correlation matrix. The variance of metabolite and transcript amounts was adjusted to equal levels by z-transformation and a pairwise correlation was subsequently undertaken. The resulting matrix revealed two prominent clusters with opposite behaviours (Figure 3). The first cluster consisted of metabolites including phenylalanine, maltose, serine, proline, raffinose and genes involved in metabolic regulation such as galactinol synthase (*GolS*), raffinose synthase (*RS*), pyrroline-5-carboxylate synthase (*P5CS*) and beta-amylase (*BMY*) which are strongly upregulated by high salinity. With respect to starch metabolism, the increase in maltose levels was positively correlated with the induction of beta-amylases (*BMY7* and *BMY8*) at the transcriptional level but negatively correlated with the amount of starch. It is worth noting that starch showed the strongest negative correlation with raffinose. The negative correlated cluster also included phosphoric acid, shikimic acid and galactonic acid.

In addition to the frequent association of metabolite levels with the expression levels of genes involved in the synthesis of these metabolites, a strong correlation between different metabolic pathways, e.g. starch mobilisation with raffinose and proline synthesis, was observed, suggesting a tight regulatory link between theses pathways in response to salt stress. Interestingly, the expression of ABA marker genes showed a strong co-regulation with numerous salt-induced metabolic changes. Transcript levels of *RAB18, ABI2* and ABA-induced *PP2C* were negatively correlated with starch contents but showed a strong positive correlation with the levels of the starch degradation product maltose, suggesting that this part of the high salinity-induced metabolic reaction might be triggered by ABA.

### Distinct and common metabolic responses to high salinity and ABA

To analyse the notion that ABA might be important for metabolic regulation and that ABA could be involved in carbon mobilisation, we studied the impact of ABA on metabolism. First, we examined the AtGeneExpress database for the transcriptional behaviour of salt-induced metabolic genes for their response to ABA. In agreement with the importance of ABA in the regulation of gene expression, the majority of the selected salinity-induced metabolic genes were also found to be upregulated by ABA (Table S4).

We next investigated the dynamic response of the *Arabidopsis* metabolome to ABA in a time course experiment. Four week old soil-grown *Arabidopsis* plants were treated with 25 µM ABA for 2 h, 6 h, 24 h and 3 d. Subsequently, the metabolic profile was determined by GC-MS (Tables S5 and S6). Comparison of the metabolic profiles of NaCl- and ABA-treated plants in an independent component analysis (ICA) revealed common and divergent metabolic responses (Figure 2A and Table S7). The first independent component (IC1) gives high loadings to metabolites with a similar behaviour in salt- and ABA-treated plants. IC1 also reflects the time response. The second independent component (IC2) distinguishes metabolites which responded differently to salt and ABA treatment. The clear separation of metabolic reactions common to high salinity and ABA treatment from those which are distinct indicates the existence of ABA-dependent and independent metabolic responses within the high salinity pathway.

Interestingly, IC2 gives a high ranking to raffinose, galactinol and G6P. Evaluation of the response of the starch to sucrose including the RFO pathway to high salinity or to ABA revealed common and stimulus-specific patterns (Figure 2B). Both high salt stress and ABA led to depletion of starch and a strong increase in maltose levels. Concordantly, beta-amylase, *BMY7*, is upregulated by both treatments. However, the next segment of the pathway responded in a manner opposite to that with salt or ABA. High salinity induced a significant decrease of the glucose to fructose ratio and a reduction of G6P levels whereas the glucose to fructose ratio (Table S2) and the G6P levels were upregulated by ABA. *HXK1* and *2* were transcriptionally induced by high salt but not by ABA.

The difference in the response to salt and ABA proceeded in the RFO pathway. While transcripts for galactinol synthase and raffinose synthase were upregulated upon both salt and ABA treatment, galactinol and raffinose accumulated under high salinity conditions but not in response to ABA. Altered carbon supply as well as post-transcriptional regulatory processes might lie at the base of these differences. The opposite behaviour of the glucose to fructose ratio and of G6P and F6P upon salt and ABA treatment indicates that a distinct carbohydrate flux might account for the deficiency of galactinol and raffinose accumulation upon ABA treatment.

Consistent with our findings, other comprehensive studies have shown that changes in transcript levels are only partially reflected in concomitant changes in mRNA translation, enzyme activities or metabolite levels [56]–[58], indicating that environmental inputs impinge on different regulatory layers and highlight that multiple layers need to be analysed to understand the complex response of plants to stress.

ABA is a central hormonal signal that regulates several aspects of stress response. With regard to metabolic adjustment to high saline conditions, ABA appears to trigger stress-induced starch mobilisation whereas salt-specific signals might be necessary for a complete metabolic adjustment to high salt. The correlation of various metabolites and genes involved in metabolic regulation with plants' sodium levels (Figure 3) suggests that the ion component of salt stress is involved in eliciting a part of the salinity-induced metabolic rearrangement. Since ABA is a key component involved in mediating different environmental stress conditions and plants respond to different stresses with overlapping but yet distinct physiological mechanisms, it is reasonable to assume that ABA-dependent signalling pathways act in concert with ABA-independent, stress-specific pathways to induce the appropriate intrinsic metabolic response.

Taken together, non-targeted and hypothesis-driven experiments were combined to identify regulatory components of salt stress signalling at the metabolite level. In this way, we discovered ABA-induced and ABA-independent steps of salt stress-induced metabolic rearrangement. A differential regulation of carbon flux or utilisation was identified upon salt and ABA treatment. Interestingly, as depicted with the RFO pathway, the expression of salt-responsive metabolic genes mainly depends on ABA while ABA only triggers a partial metabolic reaction. The presented data indicate that starch mobilisation is a fast reaction common to both high salinity and ABA-treatment. As stress-induced starch exhaustion impairs growth, identification of the ABA-responsiveness of the starch breakdown machinery now enables the identification of novel regulatory components/mechanisms and opens up new perspectives for improving yield and biomass production under unfavourable environmental conditions.

## Materials and Methods

### Plant material, plant growth and treatments

Soil grown *Arabidopsis thaliana* Col-0 plants were cultivated in an 8 h light to 16 h dark regime at 22°C, 150 µE light intensity and 60% humidity. High salinity stress was applied by watering soil-grown plants with 150 mM sodium chloride. ABA is synthesised in roots and leaves. Thus, plants were sprayed and watered with a 25 µM (+)-ABA (Sigma) aqueous solution. For prolonged ABA treatment, plants were watered and sprayed every day. For both treatments, all plants were well-turgoured and stayed green during the entire experimental period.

### Ion measurements

Soluble sodium and potassium concentrations were measured by flame photometry (atom emission spectroscopy (AES)). For this, 50 mg of pulverised plant material was extracted with 20 ml of double distilled water and the supernatant was used for analysis.

### Metabolite profiling and analysis of starch contents

Ten or fifteen rosettes of 4 week old *Arabidopsis* plants were pooled and frozen in liquid nitrogen. 60 mg of ground plant material was extracted and analysed as described in [48]. Internal standards for quantitative and retention time index calibration were added as described in [59]. 160 µl of extract was dried and stored at –80°C until metabolite analysis. The solid fraction of the extract was used for starch measurements. For this, the organic phase was removed and the pellet washed twice with 80% ethanol. The pellet was incubated with 460 µl of 0.2 M KOH at 85°C for 1 h and subsequently the pH was adjusted with 140 µl of 1 M acetic acid to pH5. 100 µl of the supernatant was incubated over night with 1 U α-amylase, 1 U β-amylase and 1 U amyloglucosidase. Free glucose was quantified using the glucose trinder kit (Sigma).

Samples for metabolite profiling were prepared as described in [48]. Derivatised extracts of salt experiment 1 were analysed with GC-TOF-MS (Pegasus III; Leco) and chromatograms were processed with ChromaTof software. Metabolites of the second salt stress experiment and the ABA treatment were analysed using a GC-Quad/MS (MD 800; Thermo), and the data were processed with the Masslab software. For peak identification, a customised mass spectral and retention time index library of ∼1000 non-redundant entries, which currently covers 360 identified metabolic components of plant, microbial and animal origin, was used [60].

### Ascorbate measurements

Ascorbate levels and the redox-state of the ascorbate pool were determined by reversed phase HPLC. 100 mg of frozen and ground plant material was extracted with 0.1 M HCl (1∶5 = material:extraction buffer) under an argon safety atmosphere. Levels of free ascorbate were analysed directly. Total ascorbate levels were determined after a reduction step with 10 mM DTT. For this, the pH was adjusted with CHES buffer (250 mM, pH9.5). Subsequently, extracts were acidified and analysed by HPLC. Separation of ascorbate was performed using an ODS column (Aquasil C18; 250 mm×4.6 mm i.d.; 5 µm; Thermo) and a Dionex HPLC system with UV detection at 254 nm. For separation, a multi-step gradient was used starting from 5% MeOH, 95% H_2_O (0–5 min) to 19% MeOH, 81% H_2_O (5–12 min) and to 90% MeOH, 10% H_2_O (12–30 min hold time 5 min). The pH of the elution buffer was adjusted to 3.9 using 0.3 M acetic acid.

### Gene expression analysis

Publicly available microarray data were generated in the frame of the AtGenExpress project (e.g. http://www.uni-tuebingen.de/plantphys/AFGN/atgenex.htm). For high salinity stress, 18 day old *Arabidopsis thaliana* Col-0 plants were treated with 150 mM NaCl. A detailed protocol for the cultivation, stress exposure and sample harvesting was recently published [11]. For ABA treatment, 7 day old *Arabidopsis thaliana* Col-0 seedlings were treated with 10 µM ABA. Samples were taken after 30 min, 1 h and 3 h [61]. Cell files were downloaded from the NASC array database (http://affymetrix.arabidopsis.info/narrays/experimentbrowse.pl) and processed using ROBIN freeware (http://www-de.mpimp-golm.mpg.de/profil/services/index.html). Expression changes of more than 4-fold were counted as relevant. Additional genes involved in the pathways of interest were included in the analyses. Transcript levels of selected genes were analysed by semi-quantitative RT-PCR. Oligonucleotide primers were designed based on gene models from the MIPS database (www.arabidopsis.com). The resulting amplicon length of the PCR products was about 120 bp. Primer sequences are listed in table S8. RT-PCR was performed from 2 µg total RNA, with oligo (dt) primers and M-MuLV Reverse Transcriptase (Q-BIOgene).

PCR was performed using MBI Fermentas Taq Polymerase according to the manufacturer's protocol. PCR products were separated on agarose gels stained with ethidium bromide.

Transcript levels were determined by reading the pixel densities using Quantity-One 4.6.1 software (BIO RAD) and normalised to tubulin used as a constitutive control.

### Bioinformatics and statistical data analyses

The statistical screening for significant metabolic changes among the monitored metabolites was performed using Microsoft Excel. PCA and ICA analysis [62], [63] were performed using MetaGeneAlyse (http://metagenealyse.mpimp-golm.mpg.de/). The ICA algorithm applied is based on 5 components of a PCA and calculates the maximal independence of non-Gaussian distributed data. The directions of variance calculated by a PCA analysis in a multidimensional room are orthogonal to each other. The ICA calculates the directions of maximal independency of the pre-analysed data set. PCA and ICA algorithms are unable to operate with missing values, thus all substances measured at all timepoints were included in the calculations.

For comparison of gene expression with metabolite data, transcript and metabolite data were z-transformed prior to pairwise correlation (Pearson correlation coefficient) analysis.

## Supporting Information

Figure S1 Soluble sodium and potassium levels in response to high soil salinity. Soluble sodium and potassium ion concentrations were determined from the same plant material that was used for metabolic profiling. 50 mg of pulverised plant material was extracted with 20 ml of double distilled water. The supernatant was measured by flame photometry (atom emission spectroscopy).
(0.01 MB PDF)

Figure S2 Principal component analysis (PCA) of technical replicas of the salt stress time course experiment shown in Figure 1. To minimize missing data points, values from 3 out of 4 replicate samples were normalised to the average of 6 control samples and used for PCA.
(0.02 MB PDF)

Table S1 Loadings of principal component analysis (PCA).
(0.05 MB PDF)

Table S2 Glucose to fructose ratio. The relative glucose and fructose contents of plants treated for 3 d with NaCl or ABA were calculated from the data presented in Table 1 and Table S3. Bold letters indicate significant changes in metabolite levels (t-test; P-value <0.05).
(0.01 MB PDF)

Table S3 Dynamic changes in metabolite levels upon high soil salinity in an independent biological experiment. Four week old soil-grown Arabidopsis thaliana was watered with 150 mM NaCl. Metabolic profiles were determined from leaves of pools of 15 individual plants four times by GC-MS. Compounds were identified by the retention time (TAG RT), the quantitative mass tag (TAG MASS) and the reverse match value (REV MATCH) of the mass spectra comparison. Starch levels were quantified spectrophotometrically. Values are x-fold changes compared to the corresponding unstressed controls. Bold letters indicate significant changes in metabolite levels (t-test; P-value <0.05).
(0.05 MB PDF)

Table S4 Transcriptional response of metabolism-related genes to high salt and ABA treatment. Transcriptional response of genes encoding enzymes in selected metabolic pathways to NaCl and ABA treatment of young, hydroponically-grown seedlings (AtGeneExpress) and adult, soil-grown plants (RT-PCR, same plant material that was used for metabolic profiling). ++, strong transcriptional induction ≥7-fold for microarray and ≥2-fold for RT-PCR; +, transcriptional induction ≥4-fold for microarray and ≥1.5-fold for RT-PCR; o, unchanged; -, transcriptional reduction ≤0.5-fold; –, strong transcriptional reduction ≤0.15-fold; −/+, transcriptional induction at late timepoints after transcriptional reduction at early timepoints; n.d., not determined.
(0.06 MB PDF)

Table S5 ABA-induced metabolic changes. Metabolite levels of 4 week old Arabidopsis thaliana after treatment with 25 µM ABA. Pools of ten plants were harvested after the indicated timepoints and the metabolite contents were analysed by GC-MS, HPLC or spectrophotometrically. Shown are the relative metabolite levels as an x-fold change normalised to untreated plants. Bold letters indicate significant changes of metabolites (t-test; P-value <0.05). TAG RT: retention time; TAG MASS: quantitative mass tag; REV MATCH: reverse match.
(0.05 MB PDF)

Table S6 Comparison of major NaCl- and ABA-induced metabolite changes.
(0.12 MB PDF)

Table S7 Table of independent component analysis (ICA) loadings.
(0.05 MB PDF)

Table S8 List of primers used for RT-PCR.
(0.05 MB PDF)

## References

[1] Boyer JS (1982). Plant productivity and environment.. Science.

[2] Hasegawa P, Bressan R, Zhu JK, Bohnert H (2000). Plant cellular and molecular responses to high salinity.. Annu Rev Plant Physiol Plant Mol Biol.

[3] Munns R (2002). Comparative physiology of salt and water stress.. Plant Cell Environ.

[4] Zhu JK (2001). Plant salt tolerance.. Trends Plant Sci.

[5] Xiong L, Schumaker KS, Zhu JK (2002). Cell signaling during cold, drought, and salt stress.. Plant Cell.

[6] Chen TH, Murata N (2002). Enhancement of tolerance of abiotic stress by metabolic engineering of betaines and other compatible solutes.. Curr Opin Plant Biol.

[7] Yancey PH (2005). Organic osmolytes as compatible, metabolic and counteracting cytoprotectants in high osmolarity and other stresses.. J Exp Biol.

[8] Gong Q, Li P, Ma S, Indu Rupassara S, Bohnert HJ (2005). Salinity stress adaptation competence in the extremophile Thellungiella halophila in comparison with its relative Arabidopsis thaliana.. Plant J.

[9] Seki M, Narusaka M, Ishida J, Nanjo T, Fujita M (2002). Monitoring the expression profiles of 7000 Arabidopsis genes under drought, cold and high-salinity stresses using a full-length cDNA microarray.. Plant J.

[10] Kreps JA, Wu Y, Chang HS, Zhu T, Wang X (2002). Transcriptome changes for Arabidopsis in response to salt, osmotic, and cold stress.. Plant Physiol.

[11] Kilian J, Whitehead D, Horak J, Wanke D, Weinl S (2007). The AtGenExpress global stress expression data set: protocols, evaluation and model data analysis of UV-B light, drought and cold stress responses.. Plant J.

[12] Christmann A, Moes D, Himmelbach A, Yang Y, Tang Y (2006). Integration of abscisic acid signalling into plant responses.. Plant Biol (Stuttg).

[13] De Smet I, Zhang H, Inze D, Beeckman T (2006). A novel role for abscisic acid emerges from underground.. Trends Plant Sci.

[14] Leung J, Giraudat J (1998). Abscisic Acid Signal Transduction.. Annu Rev Plant Physiol Plant Mol Biol.

[15] Bruzzone S, Moreschi I, Usai C, Guida L, Damonte G (2007). Abscisic acid is an endogenous cytokine in human granulocytes with cyclic ADP-ribose as second messenger.. Proc Natl Acad Sci U S A.

[16] Nagamune K, Hicks LM, Fux B, Brossier F, Chini EN (2008). Abscisic acid controls calcium-dependent egress and development in Toxoplasma gondii.. Nature.

[17] Zocchi E, Carpaneto A, Cerrano C, Bavestrello G, Giovine M (2001). The temperature-signaling cascade in sponges involves a heat-gated cation channel, abscisic acid, and cyclic ADP-ribose.. Proc Natl Acad Sci U S A.

[18] Puce S, Basile G, Bavestrello G, Bruzzone S, Cerrano C (2004). Abscisic acid signaling through cyclic ADP-ribose in hydroid regeneration.. J Biol Chem.

[19] Xiong L, Ishitani M, Lee H, Zhu JK (2001). The Arabidopsis LOS5/ABA3 locus encodes a molybdenum cofactor sulfurase and modulates cold stress- and osmotic stress-responsive gene expression.. Plant Cell.

[20] Xiong L, Lee H, Ishitani M, Zhu JK (2002). Regulation of osmotic stress-responsive gene expression by the LOS6/ABA1 locus in Arabidopsis.. J Biol Chem.

[21] Ruggiero B, Koiwa H, Manabe Y, Quist TM, Inan G (2004). Uncoupling the effects of abscisic acid on plant growth and water relations. Analysis of sto1/nced3, an abscisic acid-deficient but salt stress-tolerant mutant in Arabidopsis.. Plant Physiol.

[22] Liu X, Yue Y, Li B, Nie Y, Li W (2007). A G protein-coupled receptor is a plasma membrane receptor for the plant hormone abscisic acid.. Science.

[23] Razem FA, El-Kereamy A, Abrams SR, Hill RD (2006). The RNA-binding protein FCA is an abscisic acid receptor.. Nature.

[24] Shen YY, Wang XF, Wu FQ, Du SY, Cao Z (2006). The Mg-chelatase H subunit is an abscisic acid receptor.. Nature.

[25] Wasilewska A, Vlad F, Sirichandra C, Redko Y, Jammers F (2008). An update on abscisic acid signaling in plants and more.... Molecular Plant.

[26] Hirayama T, Shinozaki K (2007). Perception and transduction of abscisic acid signals: keys to the function of the versatile plant hormone ABA.. Trends Plant Sci.

[27] Yamaguchi-Shinozaki K, Shinozaki K (2006). Transcriptional regulatory networks in cellular responses and tolerance to dehydration and cold stresses.. Annu Rev Plant Biol.

[28] Kawasaki S, Borchert C, Deyholos M, Wang H, Brazille S (2001). Gene expression profiles during the initial phase of salt stress in rice.. Plant Cell.

[29] Kim JK, Bamba T, Harada K, Fukusaki E, Kobayashi A (2007). Time-course metabolic profiling in Arabidopsis thaliana cell cultures after salt stress treatment.. J Exp Bot.

[30] Taji T, Ohsumi C, Iuchi S, Seki M, Kasuga M (2002). Important roles of drought- and cold-inducible genes for galactinol synthase in stress tolerance in Arabidopsis thaliana.. Plant J.

[31] Kaplan F, Kopka J, Haskell DW, Zhao W, Schiller KC (2004). Exploring the Temperature-Stress Metabolome of Arabidopsis.. Plant Physiol.

[32] Cook D, Fowler S, Fiehn O, Thomashow MF (2004). A prominent role for the CBF cold response pathway in configuring the low-temperature metabolome of Arabidopsis.. Proc Natl Acad Sci U S A.

[33] Abraham E, Rigo G, Szekely G, Nagy R, Koncz C (2003). Light-dependent induction of proline biosynthesis by abscisic acid and salt stress is inhibited by brassinosteroid in Arabidopsis.. Plant Mol Biol.

[34] Handa S, Handa AK, Hasegawa PM, Bressan RA (1986). Proline Accumulation and the Adaptation of Cultured Plant Cells to Water Stress.. Plant Physiol.

[35] Bouche N, Fromm H (2004). GABA in plants: just a metabolite?. Trends Plant Sci.

[36] Bown AW, Macgregor KB, Shelp BJ (2006). Gamma-aminobutyrate: defense against invertebrate pests?. Trends Plant Sci.

[37] Fait A, Fromm H, Walter D, Galili G, Fernie AR (2008). Highway or byway: the metabolic role of the GABA shunt in plants.. Trends Plant Sci.

[38] Sanchez DH, Lippold F, Redestig H, Hannah MA, Erban A (2008). Integrative functional genomics of salt acclimatization in the model legume Lotus japonicus.. Plant J.

[39] Brosche M, Vinocur B, Alatalo ER, Lamminmaki A, Teichmann T (2005). Gene expression and metabolite profiling of Populus euphratica growing in the Negev desert.. Genome Biol.

[40] Valderrama R, Corpas FJ, Carreras A, Gomez-Rodriguez MV, Chaki M (2006). The dehydrogenase-mediated recycling of NADPH is a key antioxidant system against salt-induced oxidative stress in olive plants.. Plant Cell Environ.

[41] Tetlow IJ, Morell MK, Emes MJ (2004). Recent developments in understanding the regulation of starch metabolism in higher plants.. J Exp Bot.

[42] Lloyd JR, Kossmann J, Ritte G (2005). Leaf starch degradation comes out of the shadows.. Trends Plant Sci.

[43] Smith AM, Zeeman SC, Smith SM (2005). Starch Degradation.. Annu Rev Plant Biol.

[44] Weise SE, Kim KS, Stewart RP, Sharkey TD (2005). beta-Maltose is the metabolically active anomer of maltose during transitory starch degradation.. Plant Physiol.

[45] Khelil A, Menu T, Ricard B (2007). Adaptive response to salt involving carbohydrate metabolism in leaves of a salt-sensitive tomato cultivar.. Plant Physiol Biochem.

[46] Thimm O, Blasing O, Gibon Y, Nagel A, Meyer S (2004). MAPMAN: a user-driven tool to display genomics data sets onto diagrams of metabolic pathways and other biological processes.. Plant J.

[47] Usadel B, Nagel A, Steinhauser D, Gibon Y, Blasing OE (2006). PageMan: an interactive ontology tool to generate, display, and annotate overview graphs for profiling experiments.. BMC Bioinformatics.

[48] Kempa S, Rozhon W, Samaj J, Erban A, Baluska F (2007). A plastid-localized glycogen synthase kinase 3 modulates stress tolerance and carbohydrate metabolism.. Plant J.

[49] Geigenberger P, Reimholz H, Geiger M, Merlo M, Canale V (1997). Regulation of sucrose and starch metabolism in potato tubers in response to short-term water deficit.. Planta.

[50] Oparka KJ, Wright KM (1988). Osmotic regulation of starch synthesis in potato tubers.. Planta.

[51] Zenner R, Stitt M (1991). Comparison of the effect of rapidly and gradually developing water-stress on carbohydrate metabolism in spinach leaves.. Plant Cell Environ.

[52] Yano R, Nakamura M, Yoneyama T, Nishida I (2005). Starch-related alpha-glucan/water dikinase is involved in the cold-induced development of freezing tolerance in Arabidopsis.. Plant Physiol.

[53] Kaplan F, Guy CL (2004). beta-Amylase induction and the protective role of maltose during temperature shock.. Plant Physiol.

[54] Todaka D, Matsushima H, Morohashi Y (2000). Water stress enhances beta-amylase activity in cucumber cotyledons.. J Exp Bot.

[55] Kaplan F, Guy CL (2005). RNA interference of Arabidopsis beta-amylase8 prevents maltose accumulation upon cold shock and increases sensitivity of PSII photochemical efficiency to freezing stress.. Plant J.

[56] Gibon Y, Blaesing OE, Hannemann J, Carillo P, Hohne M (2004). A Robot-based platform to measure multiple enzyme activities in Arabidopsis using a set of cycling assays: comparison of changes of enzyme activities and transcript levels during diurnal cycles and in prolonged darkness.. Plant Cell.

[57] Kaplan F, Kopka J, Sung DY, Zhao W, Popp M (2007). Transcript and metabolite profiling during cold acclimation of Arabidopsis reveals an intricate relationship of cold-regulated gene expression with modifications in metabolite content.. Plant J.

[58] Branco-Price C, Kaiser KA, Jang CJ, Larive CK, Bailey-Serres J (2008). Selective mRNA translation coordinates energetic and metabolic adjustments to cellular oxygen deprivation and reoxygenation in Arabidopsis thaliana.. Plant J.

[59] Wagner C, Sefkow M, Kopka J (2003). Construction and application of a mass spectral and retention time index database generated from plant GC/EI-TOF-MS metabolite profiles.. Phytochemistry.

[60] Kopka J, Schauer N, Krueger S, Birkemeyer C, Usadel B (2005). GMD@CSB.DB: the Golm Metabolome Database.. Bioinformatics.

[61] Goda H, Sasaki E, Akiyama K, Maruyama-Nakashita A, Nakabayashi K (2008). The AtGenExpress hormone and chemical treatment data set: experimental design, data evaluation, model data analysis and data access.. Plant J.

[62] Scholz M, Kaplan F, Guy CL, Kopka J, Selbig J (2005). Nonlinear PCA: a missing data approach.. Bioinformatics.

[63] Scholz M, Gatzek S, Sterling A, Fiehn O, Selbig J (2004). Metabolite fingerprinting: detecting biological features by independent component analysis.. Bioinformatics.

