# Supplementary figures and images for: A Central Role of Abscisic Acid in Stress-Regulated Carbohydrate Metabolism
Source: PLoS One. 2008 Dec 12;3(12):e3935. doi: 10.1371/journal.pone.0003935 (PMC2593778; doi:10.1371/journal.pone.0003935)

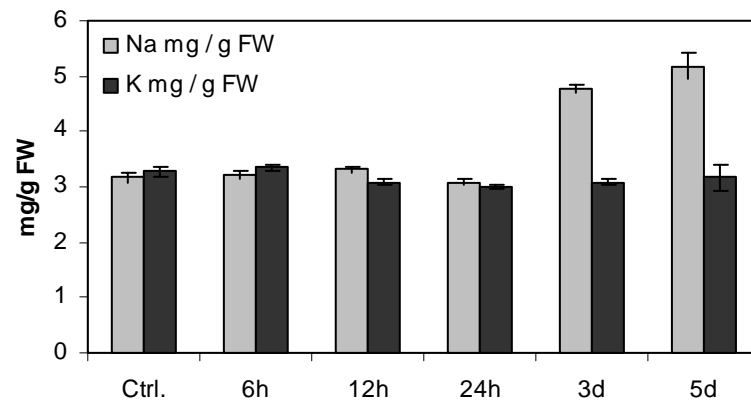

**Figure S1**

Supplement: Figure S1 — Soluble sodium and potassium levels in response to high soil salinity. Soluble sodium and potassium ion concentrations were determined from the same plant material that was used for metabolic profiling. 50 mg of pulverised plant material was extracted with 20 ml of double distilled water. The supernatant was measured by flame photometry (atom emission spectroscopy). (0.01 MB PDF) [file pone.0003935.s001.pdf]

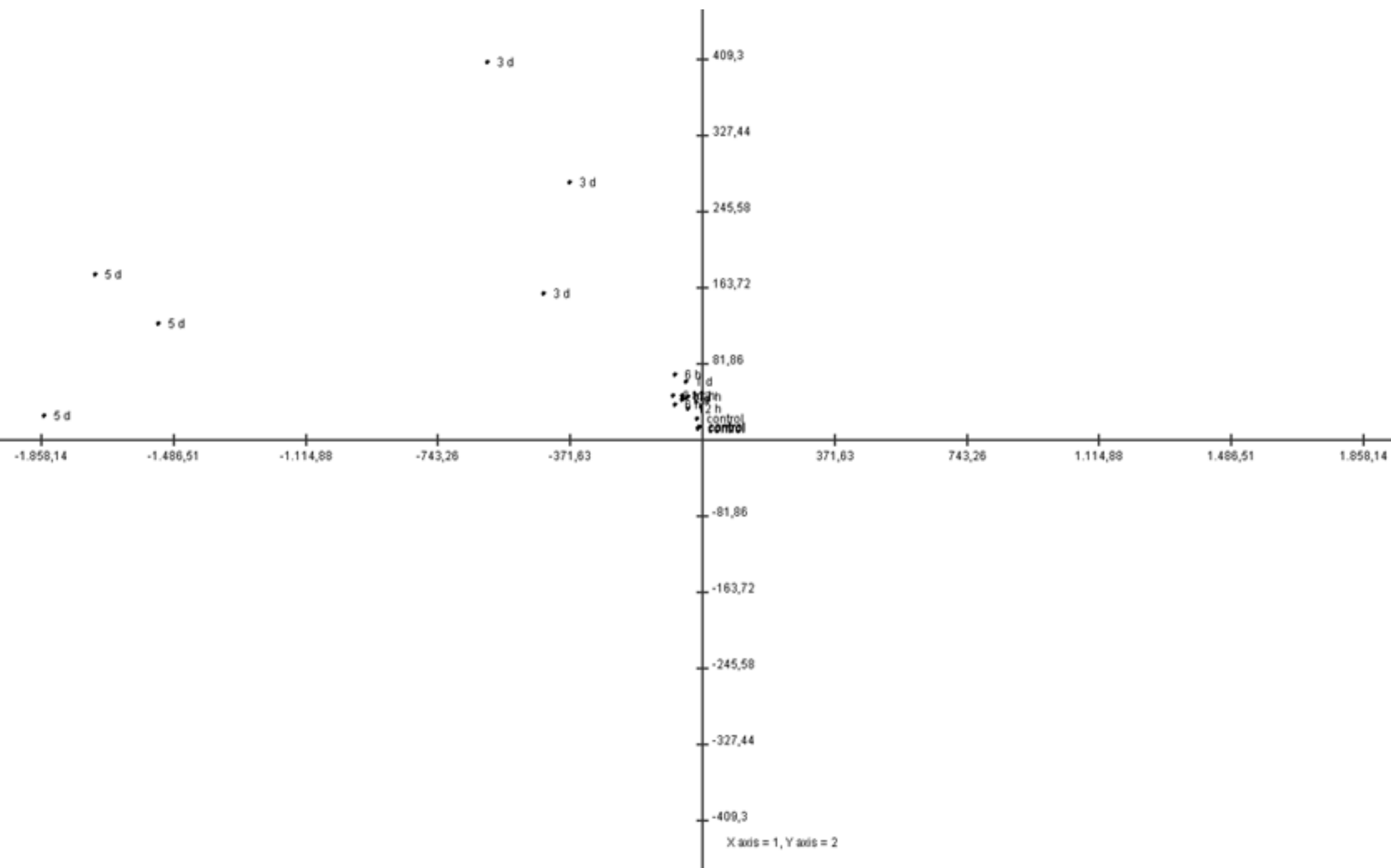

Supplement: Figure S2 — Principal component analysis (PCA) of technical replicas of the salt stress time course experiment shown in Figure 1. To minimize missing data points, values from 3 out of 4 replicate samples were normalised to the average of 6 control samples and used for PCA. (0.02 MB PDF) [file pone.0003935.s002.pdf]
