# Supplementary material for: A Central Role of Abscisic Acid in Stress-Regulated Carbohydrate Metabolism
Source: PLoS One. 2008 Dec 12;3(12):e3935. doi: 10.1371/journal.pone.0003935 (PMC2593778; doi:10.1371/journal.pone.0003935)
Supplement: Table S1 — Loadings of principal component analysis (PCA). (0.05 MB PDF) [file pone.0003935.s003.pdf]

Compound	PC1	PC2
Aconitic acid	-0.02340	0.49741
Ascorbic acid	-0.02279	0.42159
Ascorbic acid total	-0.04814	0.53171
Aspartic acid	-0.04296	0.73008
Citric acid	-0.05056	0.82380
Dehydroascorbic acid	-0.13219	0.89041
Ethanolamine	-0.07569	0.58514
Fructose	-0.14875	0.78704
Fumaric acid	-0.05430	0.76691
Galactinol	-1.34073	1.26096
Galactonic acid	-0.04420	0.48782
Gluconic acid	-0.29504	0.90136
Glucose	-0.08456	0.74229
Glucose-6-phosphate	-0.02583	0.45158
Glutamic acid	-0.15496	2.38872
Glutamine	-0.14844	0.54209
Glyceric acid	-0.04597	0.52203
Glycine	-0.39934	0.75395
Indole-3-acetonitrile	-0.08021	1.04331
Lactose	-0.04590	0.67991
Malic acid	-0.05672	0.85350
Maltose	-0.63622	5.36311
Mannitol	-0.05213	0.63755
Myo-inositol	-0.09463	0.64242
Phenylalanine	-0.35458	1.96356
Phosphoric acid	-0.02906	0.43993
Proline	-1.07250	0.93241
Pyroglutamic acid	-0.05319	0.65618
Raffinose	-6.53796	0.38985
Serine	-0.27577	3.09551
Shikimic acid	-0.03052	0.52140
Spermidine	-0.01417	0.26267
Starch	-0.02004	0.40082
Succinic acid	-0.07730	0.70421
Sucrose	-0.05741	0.62983
Threonic acid	-0.05676	0.67938
Threonic acid-1,4-lactone	-0.04067	0.54043
Threonine	-0.07833	0.89448
trans-Sinapic acid	-0.06086	0.79563
Trehalose	-0.08480	0.94184
Tyrosine	-0.50294	3.63741
Uric acid	-0.07018	0.65864
Valine	-0.28480	2.40013
γ-Aminobutyric acid	-0.93950	-0.54036

Table S1
