# Supplementary material for: A Central Role of Abscisic Acid in Stress-Regulated Carbohydrate Metabolism
Source: PLoS One. 2008 Dec 12;3(12):e3935. doi: 10.1371/journal.pone.0003935 (PMC2593778; doi:10.1371/journal.pone.0003935)
Supplement: Table S2 — Glucose to fructose ratio. The relative glucose and fructose contents of plants treated for 3 d with NaCl or ABA were calculated from the data presented in Table 1 and Table S3. Bold letters indicate significant changes in metabolite levels (t-test; P-value <0.05). (0.01 MB PDF) [file pone.0003935.s004.pdf]

	150 mM salt		25 µM ABA	
	ctrl.	***3d***	***ctrl.***	***3d***
Glucose (fold change)	1.00	**1.39**	1.00	**1.86**
Fructose (fold change)	1.00	**1.82**	1.00	1.14
Glucose/Fructose (ratio)		0.76		1.63

Table S2
