# Supplementary material for: A Central Role of Abscisic Acid in Stress-Regulated Carbohydrate Metabolism
Source: PLoS One. 2008 Dec 12;3(12):e3935. doi: 10.1371/journal.pone.0003935 (PMC2593778; doi:10.1371/journal.pone.0003935)
Supplement: Table S3 — Dynamic changes in metabolite levels upon high soil salinity in an independent biological experiment. Four week old soil-grown Arabidopsis thaliana was watered with 150 mM NaCl. Metabolic profiles were determined from leaves of pools of 15 individual plants four times by GC-MS. Compounds were identified by the retention time (TAG RT), the quantitative mass tag (TAG MASS) and the reverse match value (REV MATCH) of the mass spectra comparison. Starch levels were quantified spectrophotometrically. Values are x-fold changes compared to the corresponding unstressed controls. Bold letters indicate significant changes in metabolite levels (t-test; P-value <0.05). (0.05 MB PDF) [file pone.0003935.s005.pdf]

TAG RT	*TAG MASS*	*REV MATCH*	*COMPOUND*	*ctrl.*	*2h*	*6h*	*12h*	*24h*	*3d*	*5d*
22.22	282	320	4-Hydroxybenzoic acid	1.00	0.68	0.80	0.74	1.01	**0.50**	**0.49**
25.73	229	559	Aconitic acid	1.00	1.24	0.63	1.34	1.23	4.16	1.20
28.09	331	457	Allantoin	1.00	**1.95**	1.12	1.25	1.76	**4.43**	**2.81**
29.17	449	797	Ascorbic acid	1.00	**0.06**	0.75	0.75	**0.49**	**0.54**	0.92
32.20	381	586	Caffeic acid	1.00	0.95	1.17	1.27	1.20	0.65	1.05
29.69	381	538	cis-Caffeic acid	1.00	**2.50**	**1.70**	1.40	1.81	1.04	**1.69**
27.00	375	745	Citric acid	1.00	**0.81**	0.99	1.01	**0.79**	**0.64**	**0.51**
21.90	322	464	Cysteine	1.00	0.88	0.91	1.08	0.88	0.59	1.38
27.43	316	805	Dehydroascorbate	1.00	**1.89**	**1.45**	**1.44**	**1.62**	**0.81**	**1.57**
27.90	307	903	Fructose	1.00	**0.80**	**0.75**	1.02	**1.39**	**1.32**	**1.26**
34.72	249	332	Fructose-6-phosphate	1.00	**0.21**	0.92	1.12	**2.63**	**0.30**	0.37
17.56	147	760	Fumaric acid	1.00	**0.21**	0.85	1.11	0.73	**0.66**	0.77
43.23	361	797	Galactinol	1.00	**2.06**	0.96	**3.75**	**3.94**	**6.15**	**27.47**
29.95	333	855	Galactonic acid	1.00	0.92	1.00	1.03	**0.84**	**0.67**	0.97
28.07	466	712	Galactono-1,4-lactone	1.00	0.81	0.70	0.94	1.28	1.28	1.25
30.02	333	750	Gluconic acid	1.00	**4.63**	**1.92**	**1.94**	**2.51**	**1.46**	**1.85**
28.33	160	830	Glucose	1.00	**0.70**	**0.84**	1.07	0.94	0.29	**0.84**
34.89	299	568	Glucose-6-phosphate	1.00	**0.46**	0.77	0.93	**0.55**	**0.44**	**0.41**
23.28	363	797	Glutamic acid	1.00	**1.55**	1.15	**1.78**	**1.86**	**6.03**	**4.54**
26.17	245	796	Glutamine	1.00	1.83	1.09	2.98	3.86	1.70	**6.89**
16.38	174	811	Glycine	1.00	0.65	0.71	0.79	1.24	1.79	0.76
28.82	466	458	Gulono-lactone	1.00	0.82	0.81	1.00	0.93	**0.48**	0.81
29.28	202	202	Indole-3-acetic acid	1.00	**0.52**	1.50	0.98	0.90	1.12	**1.27**
27.17	319	409	Isocitric acid	1.00	0.72	0.68	0.54	0.76	0.90	0.77
28.75	156	373	Lysine	1.00	**1.46**	1.06	1.14	**1.41**	**10.13**	**2.47**
20.56	233	776	Malic acid	1.00	1.26	1.57	1.41	1.29	1.09	1.00
40.35	300	727	Maltose	1.00	1.00	1.27	1.24	1.10	**4.53**	**2.55**
48.88	204	155	Maltotriose	1.00	1.48	0.85	1.50	1.33	**2.25**	1.58
48.22	451	325	Melezitose	1.00	1.23	0.46	0.67	0.62	**2.60**	**2.76**
41.91	361	586	Melibiose	1.00	1.35	0.94	1.48	1.30	**3.62**	**6.64**
21.19	176	452	Methionine	1.00	**0.72**	0.87	1.02	1.11	**2.32**	**1.89**
31.46	507	869	Myo-inositol	1.00	0.87	0.96	1.12	1.03	1.21	**3.28**
26.44	308	541	O-Coumaric acid	1.00	0.79	0.95	0.80	0.74	**0.37**	**0.39**
26.93	174	368	Ornithine	1.00	0.88	1.04	**1.42**	**1.41**	**2.71**	**2.02**
23.35	266	580	Phenylalanine	1.00	**2.09**	0.84	1.48	**2.13**	**10.72**	**3.68**
15.63	314	821	Phosphoric acid	1.00	**0.45**	**0.61**	**0.72**	**0.62**	**0.23**	**0.38**
16.20	216	749	Proline	1.00	**0.62**	**0.34**	**0.65**	1.01	**12.72**	**12.71**
33.16	229	305	Putrescine	1.00	**0.38**	**0.55**	**0.64**	**0.55**	1.02	**2.13**
25.45	200	610	Putrescine	1.00	**1.65**	**0.55**	1.03	1.05	1.17	**2.28**
47.41	451	829	Raffinose	1.00	**1.59**	0.94	**3.04**	**3.05**	**12.23**	**35.33**
17.75	278	824	Serine	1.00	1.12	1.31	**1.70**	**2.23**	**5.73**	**4.60**
26.87	204	809	Shikimic acid	1.00	0.92	1.04	1.02	1.00	**0.40**	1.07
33.81	368	752	Sinapic acid	1.00	**0.54**	0.90	0.87	0.75	**0.65**	1.04
33.92	490	660	Spermidine	1.00	**0.63**	**0.71**	0.82	0.94	**0.43**	**1.72**
			Starch	1.00	0.86	1.07	1.00	0.98	**0.35**	0.99
16.68	247	816	Succinic acid	1.00	**0.66**	0.81	**0.68**	**0.72**	1.09	**0.48**
39.13	451	804	Sucrose	1.00	0.90	0.97	0.94	0.90	1.22	1.09
22.09	220	818	Threonic acid	1.00	1.02	0.94	0.90	0.79	0.81	**0.48**
18.30	291	786	Threonine	1.00	0.99	1.15	0.63	**1.54**	**2.14**	**2.41**
40.76	364	784	Trehalose	1.00	0.87	0.94	0.92	0.85	1.05	1.03
29.14	218	441	Tyrosine	1.00	0.69	1.14	1.24	1.09	**1.60**	**2.87**
32.65	456	397	Uric acid	1.00	0.76	0.75	1.12	1.15	1.47	**4.15**
21.42	174	606	γ-Aminobutyric acid	1.00	1.68	9.62	**14.52**	**26.63**	**45.76**	**15.89**

Table S3
