# Supplementary material for: A Central Role of Abscisic Acid in Stress-Regulated Carbohydrate Metabolism
Source: PLoS One. 2008 Dec 12;3(12):e3935. doi: 10.1371/journal.pone.0003935 (PMC2593778; doi:10.1371/journal.pone.0003935)
Supplement: Table S4 — Transcriptional response of metabolism-related genes to high salt and ABA treatment. Transcriptional response of genes encoding enzymes in selected metabolic pathways to NaCl and ABA treatment of young, hydroponically-grown seedlings (AtGeneExpress) and adult, soil-grown plants (RT-PCR, same plant material that was used for metabolic profiling). ++, strong transcriptional induction ≥7-fold for microarray and ≥2-fold for RT-PCR; +, transcriptional induction ≥4-fold for microarray and ≥1.5-fold for RT-PCR; o, unchanged; -, transcriptional reduction ≤0.5-fold; –, strong transcriptional reduction ≤0.15-fold; −/+, transcriptional induction at late timepoints after transcriptional reduction at early timepoints; n.d., not determined. (0.06 MB PDF) [file pone.0003935.s006.pdf]

AGI code	gene name	gene function	hydroponics 150 mM NaCl	hydroponics 10 µM ABA	soil grown plants 150mM NaCl	soil grown plants 25 µM ABA
**control genes**						
At4g25490	CBF1	CRT/CRE binding factor 1	o	+	+	+
At4g25480	CBF3	CRT/DRE binding factor 3	++	+	-/+	n. d.
At5g52300	RD29B	Desiccation-responsive protein 29B	++	++	++	n. d.
At4g23050	MAP3K	Protein kinase	++	++	++	n. d.
At1g60190	Armadillo β-catenin	Armadillo β-catenin	++	++	++	n. d.
At1g07430	PP2C	Protein phosphatase	++	++	++	n. d.
At5g59220	ABA induced PP2C	ABA induced protein phosphatase	++	++	++	n. d.
At5g57050	ABI2	ABA insensitive protein phosphatase	++	++	++	n. d.
At5g66400	RAB18	ABA responsive dehydrin	++	++	++	++
**raffinose pathway**						
At5g40390	RS	Raffinose synthase	+	+	++	+
At2g47180	GolS1	Galactinol synthase 1	++	++	++	n. d.
At1g56600	GolS2	Galactinol synthase 2	++	++	++	++
At1g09350	GolS3	Galactinol synthase 3	+	+	++	n. d.
**starch degradation and synthesis**						
At4g15210	BMY1	Beta-amylase 1	++	o	+	n. d.
At3g23920	BMY7	Beta-amylase 7	++	++	++	++
At4g17090	BMY8	Beta-amylase 8	- -	-	+	n. d.
At5g17520	MEX1	Maltose exporter	+	o	+	n. d.
At4g24450	GWD3	Glucan water dikinase	+	o	+	n. d.
At4g39210	AGPase1 (APL3)	ADP-glucose pyrophosphorylase large subunit 3	++	++	++	++
At2g21590	AGPase2 (APL4)	ADP-glucose pyrophosphorylase large subunit 4	++	++	++	++
At1g32900	St.synt.	Starch synthase	+	o	++	++
**sugar conversion**						
At5g40760	G6P DH	Glucose-6-phosphate dehydrogenase	++	o	++	++
At1g70730	PGM	Phosphoglucomutase	o	o	+	n. d.
At4g29130	HXK 1	Hexokinase 1	o	o	++	o
At2g19860	HXK 2	Hexokinase 2	+	+	++	o
At3g03250	UGPase	UDP-glucose pyrophosphorylase	+	o	o	n. d.
At3g13784	INV	Invertase	++	o	++	+
**ascorbate synthesis turnover**						
At3g47930	GL DH	Galactono-lactone dehydrogenase	o	o	o	n. d.
At1g19570	DHAR 1	Dehydroascorbate reductase 1	o	o	o	n. d.
At1g75270	DHAR 2	Dehydroascorbate reductase 2	o	o	o	n. d.
At5g16710	DHAR 3	Dehydroascorbate reductase 3	o	o	o	n. d.
At5g03630	MDHAR	Monodehydroascorbate dehydrogenase	o	o	o	n. d.
At1g77490	APX (tAPX)	Ascorbate peroxidase	-	o	-	n. d.
At3g54660	GRa	Glutathione reductase (chloroplast)	o	o	+	n. d.
At3g24170	GRb	Glutathione reductase (cytosol)	+	o	+	n. d.
At2g39770	GDP M PP	GDP mannose pyrophosphorylase	o	o	o	n. d.
**amino acids**						
At3g53260	PAL	Phenylalanine amonium lyase	-	o	++	n. d.
At1g37130	NR1	Nitrate reductase	-	-	++	-/+
At1g77760	NR2	Nitrate reductase	- -	-	+	-/+
At2g41190	GABA trans1	GABA transporter	++	++	++	++
At1g08230	GABA trans2	GABA transporter	+	++	++	+
At1g65960	GAD2	Glutamate decarboxylase	o	o	-	n. d.
At2g02000	GAD3	Glutamate decarboxylase	++	o	++	n. d.
At2g02010	GAD4	Glutamate decarboxylase	++	o	++	n. d.
At2g39800	P5CS2	Proline synthesis	++	++	++	++
At3g55610	P5CS1	Proline synthesis	++	++	++	+
At3g30775	ERD 5	Proline oxidase	- -	-	+	++
At5g38710	PrOX	Proline oxidase	o	o	+	n. d.
At1g17745	3-P glycerate DH	3-phosphoglycerate dehydrogenase	++	o	++	n. d.

Table S4
