# Supplementary material for: A Central Role of Abscisic Acid in Stress-Regulated Carbohydrate Metabolism
Source: PLoS One. 2008 Dec 12;3(12):e3935. doi: 10.1371/journal.pone.0003935 (PMC2593778; doi:10.1371/journal.pone.0003935)
Supplement: Table S5 — ABA-induced metabolic changes. Metabolite levels of 4 week old Arabidopsis thaliana after treatment with 25 µM ABA. Pools of ten plants were harvested after the indicated timepoints and the metabolite contents were analysed by GC-MS, HPLC or spectrophotometrically. Shown are the relative metabolite levels as an x-fold change normalised to untreated plants. Bold letters indicate significant changes of metabolites (t-test; P-value <0.05). TAG RT: retention time; TAG MASS: quantitative mass tag; REV MATCH: reverse match. (0.05 MB PDF) [file pone.0003935.s007.pdf]

				x-fold response (25 µM ABA)				
TAG RT	***TAG MASS***	***REV MATCH***	***COMPOUND***	***ctrl.***	***2h***	***6h***	***24h***	***72h***
22.25	282	230	4-Hydroxybenzoic acid	1.00	1.14	1.38	1.24	**1.45**
25.76	375	593	Aconitic acid	1.00	**2.52**	1.03	2.41	1.08
11.34	194	940	Alanine	1.00	1.56	**3.08**	**2.72**	**1.88**
26.95	142	474	Arginine	1.00	**2.22**	**1.52**	**2.32**	**5.07**
29.20	332	970	Ascorbic acid	1.00	**0.61**	1.07	1.58	1.03
21.20	218	872	Aspartic acid	1.00	1.27	0.73	1.85	1.49
29.03	293	203	Cinnamic acid	1.00	1.46	0.86	**5.32**	**2.89**
29.67	381	654	cis-Caffeic acid	1.00	0.78	**2.03**	4.08	1.97
27.02	375	903	Citric acid	1.00	1.09	1.07	1.13	**0.54**
21.92	322	499	Cysteine	1.00	1.50	**1.87**	**2.75**	1.49
27.46	316	946	Dehydroascorbic acid	1.00	0.99	**0.75**	**0.73**	**0.73**
27.85	307	888	Fructose	1.00	0.35	0.32	**3.82**	1.14
34.77	299	482	Fructose-6-phosphate	1.00	**2.51**	**2.51**	**2.44**	**3.15**
17.49	147	791	Fumaric acid	1.00	**0.19**	**0.11**	**0.05**	**0.07**
43.30	361	871	Galactinol	1.00	0.63	0.81	1.46	1.37
29.89	333	701	Galactonic acid	1.00	**1.74**	**2.36**	**2.55**	**3.43**
30.03	333	787	Gluconic acid	1.00	1.56	1.35	0.70	**2.04**
28.23	160	878	Glucose	1.00	0.71	0.94	**4.45**	**1.86**
34.92	299	755	Glucose-6-phosphate	1.00	**2.50**	**1.97**	**2.38**	**3.23**
23.28	246	915	Glutamic acid	1.00	**4.99**	**4.31**	**5.61**	**6.01**
26.18	245	723	Glutamine	1.00	**6.85**	3.53	**23.43**	**45.71**
16.30	248	750	Glycine	1.00	**2.03**	**1.62**	**5.76**	**3.05**
27.24	245	745	Isocitric acid	1.00	0.83	1.31	0.87	1.26
28.75	174	466	Lysine	1.00	**3.63**	**4.68**	**5.48**	**10.74**
20.61	233	955	Malic acid	1.00	1.08	1.12	**1.38**	**1.47**
40.37	300	761	Maltose	1.00	**7.70**	**2.34**	1.69	**2.61**
49.01	204	116	Maltotriose	1.00	1.11	0.72	**0.51**	0.92
48.30	451	213	Melezitose	1.00	0.79	0.78	0.78	0.89
41.92	160	514	Melibiose	1.00	0.48	0.92	1.76	2.15
21.20	176	455	Methionine	1.00	1.11	0.74	1.70	**2.73**
31.49	305	981	Myo-inositol	1.00	0.96	**0.62**	**0.79**	0.89
26.45	161	561	O-Coumaric acid	1.00	**1.51**	3.35	1.36	**2.41**
26.95	174	456	Ornithine	1.00	**2.30**	**1.75**	**2.38**	**4.45**
23.37	266	601	Phenylalanine	1.00	**2.19**	1.67	**3.99**	**10.91**
15.64	314	523	Phosphoric acid	1.00	**0.07**	**0.20**	1.28	0.64
16.15	142	656	Proline	1.00	0.47	0.55	**4.73**	**6.30**
25.46	200	781	Putrescine	1.00	0.81	1.04	**2.78**	**4.07**
10.26	174	811	Pyruvate	1.00	0.82	**0.60**	**0.45**	0.85
27.46	255	496	Quinic acid	1.00	0.98	0.91	0.67	0.90
47.50	437	913	Raffinose	1.00	**0.39**	**0.41**	0.96	**1.77**
17.75	278	947	Serine	1.00	**1.34**	1.22	**1.72**	**1.94**
26.88	462	816	Shikimic acid	1.00	**1.22**	**0.85**	0.97	0.95
33.95	490	570	Spermidine	1.00	0.86	0.67	1.29	**1.69**
			Starch	1.00	1.04	**0.66**	1.05	0.81
16.67	247	884	Succinic acid	1.00	0.79	0.60	**1.52**	0.74
39.17	451	948	Sucrose	1.00	**0.87**	**0.66**	1.03	**0.85**
22.09	292	859	Threonic acid	1.00	**2.79**	**1.90**	**1.58**	**3.39**
18.27	219	923	Threonine	1.00	0.53	0.57	0.77	0.85
31.28	381	598	trans-Caffeic acid	1.00	**3.37**	**3.43**	**5.33**	**6.65**
33.85	368	603	trans-Sinapic acid	1.00	1.24	1.16	1.34	**1.89**
40.89	364	702	Trehalose	1.00	1.04	1.02	0.96	**1.37**
29.04	218	632	Tyrosine	1.00	**2.14**	1.69	**3.43**	**6.28**
21.43	174	677	γ-Aminobutyric acid	1.00	0.90	**0.45**	**0.73**	**1.99**

Table S5
