# Supplementary material for: A Central Role of Abscisic Acid in Stress-Regulated Carbohydrate Metabolism
Source: PLoS One. 2008 Dec 12;3(12):e3935. doi: 10.1371/journal.pone.0003935 (PMC2593778; doi:10.1371/journal.pone.0003935)
Supplement: Table S6 — Comparison of major NaCl- and ABA-induced metabolite changes. (0.12 MB PDF) [file pone.0003935.s008.pdf]

	150 mM NaCl	25 µM ABA
4-Hydroxybenzoic acid	-	o
Alanine	n.d.	+
Allantoin	+	n.d.
Arginine	n.d.	+
Ascorbic acid	-	o
Cinnamic acid	n.d.	+
cis-Caffeic acid	+	+
Cysteine	o	+
Dehydroascorbic acid	+	o
Fructose	o	✷
Fructose-6-phosphate	✷	+
Fumaric acid	o	-
Galactinol	+	o
Galactonic acid	o	+
Gluconic acid	+	+
Glucose	o	+
Glucose-6-phosphate	-	+
Glutamic acid	+	+
Glutamine	+	+
Glycine	✷	+
Gulono-lactone	-	n.d.
Lysine	+	+
Maltose	+	+
Maltotriose	+	o
Melezitose	+	o
Melibiose	+	+
Methionine	+	+
O-Coumaric acid	-	+
Ornithine	+	+
Phenylalanine	+	+
Phosphoric acid	-	-
Proline	+	+
Putrescine	+	+
Pyruvate	n.d.	-
Raffinose	+	-
Serine	+	o
Shikimic acid	-	o
Spermidine	-	o
Starch	-	o
Threonic acid	o	+
trans-Caffeic acid	n.d.	+
Tyrosine	+	+
Valine	+	n.d.
γ-Aminobutyric acid	+	o

+	elevated levels (2-fold cut-off)
-	reduced levels (0.5-fold cut-off)
✷	dynamic response
o	unchanged
n.d.	not determined

Table S6
