# Supplementary material for: A Central Role of Abscisic Acid in Stress-Regulated Carbohydrate Metabolism
Source: PLoS One. 2008 Dec 12;3(12):e3935. doi: 10.1371/journal.pone.0003935 (PMC2593778; doi:10.1371/journal.pone.0003935)
Supplement: Table S7 — Table of independent component analysis (ICA) loadings. (0.05 MB PDF) [file pone.0003935.s009.pdf]

loadings for experimental points

	IC01	IC02
control	0.96	-0.32
salt 6h (exp1)	0.78	-0.10
salt 12h (exp1)	0.95	0.24
salt 1d (exp1)	0.71	-0.05
salt 3d (exp1)	-0.92	0.68
salt 5d (exp1)	-1.71	1.55
salt 2h (exp2)	1.15	0.33
salt 6h (exp2)	1.31	0.27
salt 2h (exp2)	0.55	0.41
salt 24h (exp2)	0.40	0.69
salt 3d (exp2)	-0.68	1.21
salt 5d (exp2)	-0.96	1.18
ABA 2h	0.02	-1.46
ABA 6h	0.36	-1.37
ABA 24h	-1.30	-1.71
ABA 72h	-1.62	-1.53

loadings for metabolites (sorted)

IC01		IC02	
Glutamine	-0.44	γ-Aminobutyric acid	0.46
Proline	-0.40	Raffinose	0.41
Glycine	-0.37	Glutamine	-0.38
Maltose	-0.30	Glucose-6-phosphate	-0.30
Phenylalanine	-0.28	Galactinol	0.29
Tyrosine	-0.25	Glycine	-0.22
Glutamic acid	-0.23	Galactonic acid	-0.21
Raffinose	-0.21	Threonic acid	-0.18
Glucose-6-phosphate	-0.16	Ascorbic acid	-0.15
Glucose	-0.16	Glucose	-0.15
Fructose	-0.16	Glutamic acid	-0.14
Galactinol	-0.15	Threonine	0.13
Galactonic acid	-0.13	Dehydroascorbic acid	0.13
Ascorbic acid	-0.12	Gluconic acid	0.12
Serine	-0.12	Spermidine	-0.11
trans-Sinapic acid	-0.09	Serine	0.10
Threonic acid	-0.09	trans-Sinapic acid	-0.10
Dehydroascorbic acid	0.08	Tyrosine	-0.09
Citric acid	0.06	myo-Inositol	0.09
γ-Aminobutyric acid	0.06	Aconitic acid	-0.07
Spermidine	-0.03	Phenylalanine	-0.06
Malic acid	0.03	Maltose	-0.06
myo-Inositol	-0.03	Proline	0.05
Succinic acid	-0.03	Shikimic acid	-0.05
Gluconic acid	0.03	Sucrose	0.03
Trehalose	-0.03	Fructose	0.02
Aconitic acid	-0.02	Citric acid	-0.01
Shikimic acid	0.02	Succinic acid	-0.01
Sucrose	0.01	Malic acid	-0.01
Threonine	0.01	Trehalose	0.00

Table S7
