# Supplementary material for: A Central Role of Abscisic Acid in Stress-Regulated Carbohydrate Metabolism
Source: PLoS One. 2008 Dec 12;3(12):e3935. doi: 10.1371/journal.pone.0003935 (PMC2593778; doi:10.1371/journal.pone.0003935)
Supplement: Table S8 — List of primers used for RT-PCR. (0.05 MB PDF) [file pone.0003935.s010.pdf]

AGI code	gene name	gene function	RT primer fwd 5'-3'	RT primer rev 5'-3'
**control genes**				
At4g25490	CBF1	CRT/CRE binding factor 1	ACGTGTCGCTTTGGAGTTAC	GAAGGAACCATTCTAAAAAAAGGAAC
At4g25480	CBF3	CRT/DRE binding factor 3	AATCATGAAGTCGACGGCGATG	TGAAGAATCCATTCTAAAAAAAGATC
At5g52300	RD29B	Desiccation-responsive protein 29B	GGAGTTCAAGATTCTGGGAAC	CATCAAAGTTCACAAACAGAGGC
At4g23050	MAP3K	Protein kinase	TGAGCCTGTTCCGCAAACGCA	CACGTCCTTTGCTTTGTGAAC
At1g60190	Armadillo β-catenin	Armadillo β-catenin	ACCGGTTCTAGAAAGAGAACG	GCTCAAAGAGTTATTGTGTATCATC
At1g07430	PP2C	Protein phosphatase	CACAGTAGTGACAACGTAAGCG	TGGAGTAAGAGTGATGAGGAG
At5g59220	ABA induced PP2C	ABA induced protein phosphatase	GTTCGGACAACGTAAGTGTTG	CAAAAGTCGATGACTCAGGAC
At5g57050	ABI2	ABA insensitive protein phosphatase	GAAGGGAATAAGGAAATTCAAGAGC	AAGAGAATTTTTACCCACCATG
At5g66400	RAB18	ABA responsive dehydrin	AAGGAAAAGCTTCCCGGTG	CGACTGCGTTACAAACCCTC
**raffinose pathway**				
At5g40390	RS	Raffinose synthase	CAAGTCCCTTGGTCTGGTCC	AACTCAACGAACGGTACGGAG
At2g47180	GolS1	Galactinol synthase 1	CAACTACGTGAACGCACCG	CATAATCCCCAACCTCTTACTG
At1g56600	GolS2	Galactinol synthase 2	CTTTGTCTGAAGCCGGTGCG	TAGTCATGAAGAGGCGTATGC
At1g09350	GolS3	Galactinol synthase 3	TGTCTGAAGCTGATGTGCTTC	CACCAGTTTCACAAACTATTAGC
**starch degradation and synthesis**				
At4g15210	BMY1	Beta-amylase 1	GAGCATTTAAGTGGGACTCTG	CTTACTTCCTTGATTCTTTCCC
At3g23920	BMY7	Beta-amylase 7	GCTGGTTCAAGAAGCTGCAG	GTCATATAACCATTTCATTATAATCGC
At4g17090	BMY8	Beta-amylase 8	AAGGCAAGATCGCTGAGAATG	CACTCTTACTGGTTTGATCTCTC
At5g17520	MEX1	Maltose exporter	GCTTATGGTCACAACTCGCC	CTCTATGGACTTGCTTGAACAAAGG
At4g24450	GWD3	Glucan water dikinase	CCTCAAGACATTGAAGGTGTTG	GATTTGATTGAAAACTTAGGGAATGG
At4g39210	AGPase1 (APL3)	ADP-glucose pyrophosphorylase large subunit 3	GATCTGGAATCACTGTGGTTG	AACTTCCGGCCAAACACCTCAAAATC
At2g21590	AGPase2 (APL4)	ADP-glucose pyrophosphorylase large subunit 4	CTACATTCGATCAGGAATCACTG	GATTGTTGCTCGGTGGACTTAC
At1g32900	St.synt.	Starch synthase	GTGAAGAGATAGCTCCTCTG	CGCTTTCTTCAAACCACTTTGAC
**sugar conversion**				
At5g40760	G6P DH	Glucose-6-phosphate dehydrogenase	AGCTGGTTATCTTCAGACTCAC	ACTCGAGGAGGGACTATTCAG
At1g70730	PGM	Phosphoglucomutase	GCTGTCGAAAATGCAAGAGTTC	CCAAATTCATAAGAAATGAAGTTG
At4g29130	HXK 1	Hexokinase 1	GCTTCTTGCTGCTTCTCACTC	CTTAGGCGTTTTCTGATAGCG
At2g19860	HXK 2	Hexokinase 2	CTTCTCACTCTCAGTATCTCG	CTAACGGCAAAACAAAGAAGAAGG
At3g03250	UGPase	UDP-glucose pyrophosphorylase	AATGGTCCAGAAGACCTCTG	CATGAGAGAGGGAAGAAGAC
At3g13784	INV	Invertase	AGCCTAAGTGCTTGGAGCATG	GTGAAAGAGGAGCTTCTTTATATTC
**ascorbate synthesis turnover**				
At3g47930	GL DH	Galactono-lactone dehydrogenase	ATCCTCTCCAACAACATGGTG	GATGTATAGGATCCCAAAAAGAGG
At1g19570	DHAR 1	Dehydroascorbate reductase 1	TTCAACCAAGCACCACCGCC	AACCCTTCCCGGTTTAGTCGAAG
At1g75270	DHAR 2	Dehydroascorbate reductase 2	GAATGCGTGATGTGGATCCAT	GAACACACACATACTCGTGC
At5g16710	DHAR 3	Dehydroascorbate reductase 3	CTATGCACCGTTATGAGATCG	AAAGGTCCAAGTACATTATTTGACTG
At5g03630	MDHAR	Monodehydroascorbate dehydrogenase	AAAGGTCCAAGTACATTATTTGACTG	GGGTCAAACAAATAACAAAAATGTCG
At1g77490	APX (tAPX)	Ascorbate peroxidase	TTGATTTCTCTTACCTACATACAT	CAAGCAAACTTTAACAATGATGT
At3g54660	GRa	Glutathione reductase (chloroplast)	ATGGCTTCGACCCCGAAGCTTACC	TAGGAAGCGAGAGAAAGCTAGAGG
At3g24170	GRb	Glutathione reductase (cytosol)	ATGGCGAGGAAGATGCTTGTTG	CACTCCCGGCACCGATGAC
At2g39770	GDP M PP	GDP mannose pyrophosphorylase	CAATGGAGGAGTTGTTTTGCC	GCGATTTCAAAGTTGAAGAAAGG
**amino acids**				
At3g53260	PAL	Phenylalanine amonium lyase	GATCCGTTGATGGATTGTCTC	AAACTCAATTCAAACATTAACACAGC
At1g37130	NR1	Nitrate reductase	GTTTGCGGTTCAGCCGAATTTG	GCTTTTTGACGACTTAACGTTTGAC
At1g77760	NR2	Nitrate reductase	AGTTTGCGTTGCAGCCAAATC	CACATAAGCGATACTTGTTATCAAC
At2g41190	GABA trans1	GABA transporter	GTGGCAAAGATCATTAGGAAC	ACAGGGGATAAACTATGGGTC
At1g08230	GABA trans2	GABA transporter	GACAGATTATAATAGATGCCAATACG	GGCTTATTCTTAAATATAGAAATAAC
At1g65960	GAD2	Glutamate decarboxylase	CTGATGGAAGTTATTGTTGGATGG	CTGATAAACGCAAAGTCCTCGCAAG
At2g02000	GAD3	Glutamate decarboxylase	CAAGAACGGCGTTCCGTTAG	TGGTGTAGTTGGGCCTTTAGC
At2g02010	GAD4	Glutamate decarboxylase	CTGGAAGAAGTTGTTGGAGAC	AAGGTCCATAATTATGGAGCGC
At2g39800	P5CS2	Proline synthesis	ATTGTTTACACCCATCAGGAC	TCTGTGTCTCTCCTCAAGTC
At3g55610	P5CS1	Proline synthesis	ACCCATAAGGATCTTCCTGTA	CCTCAAGGAACACTATATCCTATG
At3g30775	ERD 5	Proline oxidase	GGAACTTAAGAGGAGATTAATCG	TTCAATTTCTTAAACTACATAGAAG
At5g38710	PrOX	Proline oxidase	GGAGCTTAAGAGAAGAGTTATGG	CATTTGTGGATATTTTGCTACTCC
At1g17745	3-P glycerate DH	3-phosphoglycerate dehydrogenase	TGTTTCTGCGATTGAAGAGTTTG	TAACAACCACTGGATAAAACACC

Table S8
